# Dissociating the Multiple Psychological Processes in Everyday Moral Decision-Making with the CAN Algorithm

**DOI:** 10.3390/bs12120501

**Published:** 2022-12-08

**Authors:** Zhongju Xie, Junhong Wu, Xingyuan Wang, Ziyi Zheng, Chuanjun Liu

**Affiliations:** 1 Department of Sociology and Psychology, School of Public Administration, Sichuan University, Chengdu 610065, China; 2 School of Economics, Sichuan University, Chengdu 610065, China; 3 Institute of Psychology, Sichuan University, Chengdu 610065, China

**Keywords:** everyday moral decision-making, CAN algorithm, process dissociation, altruistic, egoistic

## Abstract

In previous research frameworks, researchers used an everyday dilemma to test people’s altruistic versus egoistic inclination. However, there are at least three different psychological processes that could induce altruistic over egoistic decisions, i.e., stronger altruistic sensitivity, weaker egoistic sensitivity, and stronger overall action versus inaction preference. To dissociate these different psychological processes, we developed new materials and applied the CAN algorithm from traditional moral dilemma research in two studies. In Study 1, we designed scenarios varying with a 2 (egoistic/non-egoistic) × 2 (non-altruistic/altruistic) structure. Then, we recruited 209 participants to validate the scenarios and filtered six scene frameworks with 24 scenarios in total. In Study 2, we recruited 747 participants to judge whether they would conduct behavior that is simultaneously altruistic (or non-altruistic) and egoistic (or non-egoistic) in the filtered scenarios obtained from Study 1. They also filled in the Social Isolation Scale, Distress Disclosure Scale, and some other demographic information. As we dissociated the psychological processes using the CAN algorithm, significant correlations between social isolation and distress disclosure and three parameters (i.e., altruistic tendency, egoistic tendency, and overall action/inaction preference) underlying the altruistic choice were revealed to varying degrees. Other individual differences in the psychological processes in everyday moral decision-making were further demonstrated. Our study provided materials and methodological protocols to dissociate the multiple psychological processes in everyday moral decision-making. It promotes our insights on everyday moral decisions from a differential psychological processes perspective.

## 1. Introduction

Consider the following situation. The community you live in required residents to stay at home and quarantine, considering the obvious safety concerns during the COVID-19 pandemic. One day, you find a note asking for help. A neighbor wants some meat for his/her children. If you give this neighbor the only piece of meat left in your home, he/she will be able to alleviate the shortage. However, you will only have noodles to eat for the next few days. In this situation, would you give your meat to the neighbor?

In daily life, you may encounter a conflict situation that benefits others but is bad for yourself (or benefits yourself but is bad for others), and you must make a behavioral choice between altruism and egoism. It is known as everyday moral decision-making [1]. If you tend to choose altruism, it might be because you have a stronger altruism sensitivity and you really want to benefit others. Alternatively, it could also be because you have a weaker egoism sensitivity, and you do not mind sacrificing your own interests. Furthermore, it might also be due to your general responding preference, which means a stronger acceptance bias to do everything ignoring the nature (altruistic or egoistic) of the request. These potential psychological processes could not be dissociated in previous research paradigms of everyday moral decisions, and the present study aims to fill this research gap and develop materials for dissociating the multiple psychological processes in everyday decision-making.

### 1.1. Learning Research Paradigms from Traditional Moral Dilemma Research

Everyday moral dilemma has a similar conflicted structure as the traditional moral dilemma, which enlightens us to borrow research paradigms from traditional moral dilemma research. Previous research on moral decision-making has often been conducted in the context of abstract moral dilemmas, such as the trolley dilemma (i.e., deciding whether to allow a runaway trolley to kill five people or to change the direction of the trolley’s trajectory to kill one person and save the five [2]) and the footbridge dilemma (i.e., the only way to save five people from a runaway trolley is to block the trolley by pushing someone off a pedestrian bridge [3]). Prior researchers concluded that people have more utilitarian inclination or weaker deontological inclination if they tended to agree with sacrificing one innocent life to save five. However, it is ambiguous to interpret the effect of greater willingness to sacrifice. People might have a stronger utilitarian inclination and care more about the beneficial result of saving five over sacrificing one (i.e., being sensitive to consequences). They might also have a weaker deontological inclination and care less about the harming nature of the sacrificing behavior (i.e., being sensitive to norms). Moreover, they might have a general acceptance bias to conduct any behaviors ignoring the results and the nature of the behavior (i.e., having general inaction/action preferences). To dissociate these possibilities, Gawronski et al. [4] developed a multinomial processing model to measure the above-mentioned consequence sensitivity (depicted by C parameter), norm sensitivity (depicted by N parameter), and generalized inaction/action preference (depicted by I parameter), named the CNI model. 

The CNI model is constituted of two parts, structured dilemma materials and parameter estimation method. For the structured dilemma materials, Gawronski et al. [4] developed six moral dilemmas based on previous research and the incidents happening in the real world. Each dilemma is manipulated by the consequences (benefits are greater/smaller than costs) and nature (the behavior is advocated/prohibited by moral norms) of the behavior. Thus, each dilemma has four parallel versions, and participants were asked whether they would conduct such behaviors in the given scenario [4]. These materials provide a basis for estimating the parameters. Parameters can be estimated by comparing the different responses to these four parallel versions of dilemmas. For the parameter estimation method, they applied the multinomial processing paradigm and used the hierarchical maximize likelihood estimation approach. The parameter can be estimated at the group level [4] and also at the individual level [5].

Although the CNI model [4] contributes to dissociating the multiple psychological processes of traditional dilemma decision-making, it is based on the premise that people’s decision processing takes place in a sequential manner. It is assumed that individuals make decisions based on consequences first and norms second. Once neither consequences nor norms are considered, people will make a general acceptance or rejection response. This approach ignores the possibility of other parallel processing [6,7]. Liu and Liao [7] proposed the CAN algorithm, which calculates the corresponding parameters (i.e., consequence sensitivity, norm sensitivity, and overall action/inaction preference) by evaluating the decision maker’s acceptance or nonacceptance of the behavior proposals in the provided material. Specifically, four probability data (i.e., p1, p2, p3, and p4) exist for each of the four parallel versions of dilemmas, representing the decision maker’s averaged degree of approval for the proposed behavior in that type of dilemma. The CAN algorithm works as a linear approach to generating the parameters and overcomes some limitations of the CNI model [8].

To summarize, we need to learn two things if we want to borrow the research paradigm from the traditional moral dilemma decision-making. One is the development of the structured dilemma material, and the other is the method of parameter estimation.

### 1.2. Learning Point 1: Structured Dilemma Scenarios Development

Before we discuss how to compile the structured dilemma scenarios, we briefly reviewed the scenario materials that existed for everyday moral decision-making. Traditional moral dilemmas require moral reasoning about extreme cases of life or death. It is difficult to generalize these dilemmas to everyday life because of the lack of external and ecological validity [9]. 

Attempts have been made to explore people’s everyday moral decision-making through vignettes describing hypothetical everyday situations [10]. Starcke et al. [11] subdivided high-mood and low-mood dilemmas in their measurement materials, but a problem remains that some high-mood dilemmas are not common life situations (e.g., deciding to leave a suicidal partner). Sommer et al. [12] developed 56 stories for examining neural links in everyday moral decision-making. Their material was divided into two main categories, namely, moral conflict and neutral stories. Participants were asked to make a choice between personal desires and moral standards (i.e., moral conflict situations) and conflicting personal desires (i.e., neutral situations). However, these materials yielded similar limitations to traditional moral dilemmas in that they had a single-dimensional structure. That is, once participants choose altruistic behaviors, they are considered non-egoistic, which is somewhat ambiguous in terms of theoretical interpretation [7]. 

The fact that people choose more altruistic behaviors does not necessarily mean that they have a stronger tendency to be altruistic. It is also possible that they simply have weaker egoistic tendencies or that they have a stronger tendency to accept the given behaviors without considering the altruistic or egoistic nature of the behavior at all. The previous model of decision-making that opposed altruistic and egoistic tendencies could not effectively dissociate these possibilities.

Learning from the traditional dilemma research, we can develop four types of everyday moral decision-making dilemmas (egoistic and non-altruistic, egoistic and altruistic, non-egoistic and altruistic, and non-egoistic and non-altruistic), as shown in Table 1. From the same background, there are four different structured situations with 2 (egoistic/non-egoistic) × 2 (non-altruistic/altruistic). We can compare the different responses in these four parallel versions of scenarios to estimate whether people are sensitive to altruism or egoism and whether they generally tend towards action/inaction. To make the estimation more stable and reliable, we need to compile more parallel versions of scenarios. Considering the energy costs for the participants, we did not present too many scenarios to them in order to avoid fatigue. Six scene frameworks with four parallel versions for each are recommended, according to previous research [4].

### 1.3. Learning Point 2: Parameter Estimation Method

As discussed in Section 1.1, the estimation method of the CNI model is optimized and replaced by the linear method of the CAN algorithm [7]. In present study, we decide to learn and apply the CAN algorithm to quantify the multiple psychological processes in everyday moral decision-making. 

Following the basic logic of the CAN algorithm, the meanings represented by p1, p2, p3, and p4 are as follows:

- p1: The probability of doing an act that is egoistic and non-altruistic when faced with the egoistic and non-altruistic scenarios.
- p2: The probability of doing an act that is egoistic and altruistic when faced with the egoistic and altruistic scenarios.
- p3: The probability of doing an act that is non-egoistic and altruistic when faced with the non-egoistic and altruistic scenarios.
- p4: The probability of doing an act that is non-egoistic and non-altruistic when faced with the non-egoistic and non-altruistic scenarios.

As there are multiple scenarios in each of the four parallel versions, participants might agree with the behavior in some of the scenarios and disagree with the behavior in the other scenarios. Further, we can calculate the probability of agreeing to conduct the behaviors in each type of the scenarios, i.e., p1, p2, p3, and p4 (as shown in Table 1).

According to the above four probabilistic indicators, three parameters representing individuals’ everyday moral decision-making tendencies can be generated according to the CAN algorithm [7] and the follow-up studies [13,14]. We named the parameters as follows:

- Altruistic Tendency (AT) = (p2 − p1 + p3 − p4)/2.
- Egoistic Tendency (ET) = (p2 − p3 + p1 − p4)/2.
- Overall Action/Inaction Preference (OP) = (p1 + p2 + p3 + p4)/4.

The three parameters portray the multiple psychological processes that people engage in when making moral decisions in everyday life. The altruistic tendency (AT) refers to the extent to which people make decisions based on the principle of benefiting others; the egoistic tendency (ET) refers to the extent to which people make decisions based on the principle of benefitting themselves; the overall action/inaction preference (OP) refers to the extent to which people have an overall tendency to conduct behaviors, regardless of the specific situation in which the behavior is proposed.

### 1.4. Current Study

As discussed above, we aimed to learn research paradigms from traditional moral dilemma decision-making and to develop the materials to dissociate the multiple psychological processes in everyday moral dilemma decision-making. In Study 1, we analyzed learning point one, i.e., developing the structured scenarios. Further, we needed to test whether participants agree with our structural design. If we designed the scenario to be non-egoistic and altruistic (i.e., the scene p3 in Table 1), we needed to test whether participants agree that the behavior in this version is non-egoistic and altruistic. Otherwise, that participants have different responses to the four parallel versions might not be because of the different altruism and egoism sets but because of their different understanding towards these scenarios. This kind of response pattern was called perverse responses [6]. To avoid this type of confound and test the construal and structural validity of our materials, we conducted Study 1 and predicted that the participants have the consistent evaluations to our hypothesized structure (Hypothesis 1).

In Study 2, we analyzed learning point two and gave examples of how this new method dissociates the multiple psychological processes in everyday moral dilemma decision-making. Given the background of COVID-19, we mainly considered the relationships between altruism and two variables, namely, social isolation and distress disclosure. 

Altruism means a behavioral tendency of an individual, at least in part, to benefit others at a cost to oneself [15]. Previous research has typically emphasized the positive correlation between altruism and social integration or social connection [16,17]. However, if individuals feel out of step with others (i.e., develop a sense of social isolation), they may instead engage in more altruistic behaviors as a result. Social isolation refers to an individual’s perceived social distance from others [18]. This sense may be associated with poorer health quality of life, lower life satisfaction, and less social engagement [19,20,21]. Additionally, socially isolated individuals lack opportunities to share their feelings with significant others [22]. In this case, they may have a stronger motivation to restore social connectedness as belongingness is an example of a generally valued goal. Altruism may play an important role in the achievement of this goal. Therefore, from restoring the social connectedness perspective, the present study hypothesized that social isolation may be positively associated with an individual’s altruistic tendency in everyday moral decision-making (Hypothesis 2). We left an open hypothesis that this positive correlation might be decomposed into different correlations between social isolation and the multiple psychological processes underlying everyday moral decision-making.

Altruistic tendencies may be tied to distress disclosure in the background of COVID-19. Distress disclosure is a subset structure of self-disclosure, which means that one person expresses his/her feelings and beliefs honestly to others [23]. Self-disclosure is the disclosure of a broad aspect of the self, and distress disclosure requires the revealed information to be focused on the individual’s unpleasant thoughts or feelings [24]. Therefore, disclosing unpleasant emotions and events to others may improve people’s mental state. The research on the outcomes of distress disclosure also supported its positive mental health consequences for people (e.g., the increase in happiness, the decrease in global psychological symptoms and perceived stress, and the decrease in symptom distress and social-role difficulties [25]). Once the mindset had broadened, an individual may shift the attention from himself/herself to others. For example, individuals with higher levels of well-being were found to do more volunteering [26]. Thus, this study hypothesized that there may be a positive correlation between distress disclosure and altruistic tendencies (Hypothesis 3). We also leave an open hypothesis that this positive correlation might be decomposed into different correlations between distress disclosure and the multiple psychological processes underlying everyday moral decision-making.

To sum up, the present study aimed to develop and validate materials for assessing people’s multiple psychological processes in everyday moral decision-making. Additionally, we will examine the association between social isolation, distress disclosure, and these multiple psychological processes.

## 2. Study 1: Development and Validation of Scenarios for the Psychological Process Dissociation in Everyday Moral Decision-Making

In the moral dilemmas of decision-making, people may not necessarily have a higher altruistic tendency even if they choose to behave more altruistically. It is possible that they have weaker egoistic tendencies or stronger overall action/inaction preferences. This idea of decision-making in a parallel path originated from Liu and Liao [7]. According to them, when making decisions with dilemmas, decision makers consider both norms (prohibited or advocated) and outcomes (benefits greater than costs or benefits smaller than costs), and they are influenced by the interaction of both principles. Accordingly, Study 1 developed factorial structured scenario materials to dissociate altruistic tendency in everyday moral decision-making with reference to structured scenarios designed by Gawronski et al. [4] and the latest CAN algorithm [7]. Before applying these materials, the consistency between the designed structure and people’s judgment of the structure needs to be tested. For example, when the researchers designed a scenario that was non-egoistic and altruistic, we need to test whether participants also perceived the scenario as non-egoistic and altruistic. In this way, the construct validity of the materials can be ensured.

### 2.1. Materials and Methods

#### 2.1.1. Participants

Participants were recruited through Wenjuanwang, a Chinese professional survey website. All participants completed an informed consent form on the home page of the electronic questionnaire and authorized the researcher to use their data for teaching and research purposes. All participants received a monetary reward of 3 RMB for participating. 

A total of 209 responses were obtained after excluding samples that failed the instructional manipulation check [27]. The age of the sample ranged from 16 to 69 years old (M_age_ = 27.9 years, SD = 7.47 years, 27.3% males). A total of 89 (42.6%) participants were single, and 120 (57.4%) participants were in an intimate relationship.

#### 2.1.2. Development of Materials

The materials developed by the authors consist of 13 everyday life situations frameworks. Each framework contains four types of dilemmas (egoistic and non-altruistic, egoistic and altruistic, non-egoistic and altruistic, and non-egoistic and non-altruistic) with a total of 52 scenarios.

Each scenario ended with a proposed behavior, as shown in Table 1. Participants were asked to judge whether the outcome of the behavior is, (A) good for yourself and bad for others, (B) good for others and bad for yourself, (C) good for both yourself and others, and (D) bad for both yourself and others. The participants were instructed to choose only one answer for each of the 52 scenarios.

#### 2.1.3. Procedure

The electronic questionnaire is titled Judgement of Scenarios. All participants filled out an informed consent form on the first page of the questionnaire and then read 52 scenarios and performed the judgment task. The task was introduced as “You will read a number of short scenarios in which you need to make judgments about the benefits and costs of the actions in the scenarios for yourself and for others”. The order of presentation of the material was pre-randomly controlled to ensure that two adjacent segments were from different dilemmas. Question 40 was used to check instructional manipulation (i.e., We need to confirm that the respondent has read the materials carefully and made judgments based on the provided materials as much as possible. Therefore, this question is an instructional manipulation check. Please select options A., B., C., and D.). If the respondent did not pick a specific option as instructed, the response was considered invalid and will not be involved in the subsequent analysis.

#### 2.1.4. Analytical Strategy

After the questionnaire was collected, the participants who passed the instructional manipulation check were screened out. All valid data were imported into SPSS 25.0 for coding. If the options selected by the participants were consistent with the type of scenario assumed by the authors (e.g., when the authors designed a scenario that was non-egoistic and altruistic, participants also perceived the scenario as non-egoistic and altruistic), the answer was encoded as 1, otherwise it was coded as 0. A chi-square test was subsequently conducted to test whether there is higher frequency of code 1 than code 0 for each of the scenarios.

### 2.2. Results

As shown in Table 2, among the 52 scenarios, there were 14 scenarios in which participants’ judgments failed to be significantly more consistent with the intended structure than inconsistent evaluations, and some scenarios had significantly opposite results. In order to maximize the material’s conformity to the intended structural design, the scene framework to which these scenarios belonged was excluded as a whole in this study. A frame was retained only if the percentage of participants who judged all four-dimension structures to be consistent with the design was significantly or marginally significantly higher than 50%. Finally, six scene frameworks (i.e., a friend asks to board a pet at your home, a relative asks you to be a tour guide for a trip, a colleague faces an examination, a stranger is smoking in public, a salesperson asks you to sign up for a membership, an online purchase needs your evaluation) with a total of 24 scenarios were obtained (see Appendix A Table A1 and Table A2).

### 2.3. Discussion

Sommer et al. [12] pointed out that everyday moral dilemmas describe short segments of hypothetical everyday life. This entails a choice between personal hedonism that does not cause serious physical harm or legal consequences (individual-oriented, namely, egoism) and fulfilling moral obligations to others (other-oriented, namely, altruism). Therefore, the present study designed a factor structure to measure individuals’ altruistic and egoistic tendencies. The scenarios are all derived from daily life and have high ecological and external validity, which can be used as material for investigating everyday moral decision-making.

The 24-item material developed by Gawronski et al. [4]—consisting of six moral dilemmas, each containing four dimensions obtained by the intersection of norms (prohibited/advocated) and consequences (benefits greater than costs/benefits smaller than costs)—provides a reference for this study. With a structured setup of 2 (egoism: egoistic/non-egoistic) × 2 (altruism: altruistic/non-altruistic), we developed four types of everyday moral decision-making dilemmas (egoistic and non-altruistic, egoistic and altruistic, non-egoistic and altruistic, and non-egoistic and non-altruistic) with a total of six scene frameworks. The hypothesis 1 is partly supported. It was shown in Study 1 that we obtained six frameworks with 24 scenarios. In these scenarios, participants have significantly consistent evaluations of the altruistic/egoistic nature of the proposed behavior.

## 3. Study 2: Example of Dissociating the Multiple Psychological Processes in Everyday Moral Decision-Making: Exploring the Relationships between Altruistic Tendencies and Social Isolation and Distress Disclosure

### 3.1. Materials and Methods

#### 3.1.1. Participants

As in Study 1, participants were recruited through the website named Wenjuanwang. All participants completed an informed consent form on the home page of the electronic questionnaire and authorized the researcher to use their data for teaching and research purposes. All participants received a monetary reward of 3 RMB for participation. 

A total of 747 responses were obtained after excluding samples that failed the instructional manipulation check [27]. Subsequently, samples with response latency outside of three standard deviations were excluded. The final sample consisted of 734 cases, aged from 16 to 66 years old (M_age_ = 26.66 years, SD = 6.53 years, 42.1% males). A total of 358 (48.8%) participants were single, and 376 (51.2%) participants were in an intimate relationship.

#### 3.1.2. Measurements

- Social isolation. The 6-item Friendship Scale (FS), developed by Hawthorne [18], was used to assess the perceived social distance from others (e.g., When with other people I felt separate from them). It was rated on a five-point Likert scale (from 1 = strongly disagree, to 5 = strongly agree), with three reverse coded items. The higher the total score, the stronger the feeling of social isolation. The original version of the scale was in English and was translated into Chinese under the guidance of a teacher majoring in English. The McDonald’s ω coefficient was 0.82 and the Cronbach’s alpha coefficient for the scale in this study was 0.81.

Convergent validity was assessed through factor loading, composite reliability, and average variance extracted by the constructs. As shown in Table 3, five items with factor loading larger than 0.5 were retained. The composite reliability (CR) value of FS was 0.82, exceeding the recommended level of 0.70 [28]. The average variances extracted (AVE) of FS was 0.48, close to the cut-off value of 0.5 [29]. The following measured indices was assessed for overall model fit. A good model fit was found for this scale in the confirmatory factor analysis (CFA): *χ*^2^/DF = 0.684, *p* = 0.603, GFI = 0.999, AGFI = 0.994, CFI = 1.000, TLI = 1.002, IFI = 1.001, RMSEA = 0.000 [0.000, 0.047], and SRMR = 0.008.

- Distress disclosure. The 12-item Distress Disclosure Index (DDI; [30]) was used. Participants were asked to rate on a five-point Likert scale (from 1 = almost, to 5 = not at all) their feelings about each item (e.g., When something unpleasant happens to me, I often look for someone to talk to). Six of the items require reverse scoring. Higher scores indicate a higher level of distress disclosure. The original version of the scale was in English and was translated into Chinese under the guidance of a teacher majoring in English. The McDonald’s ω coefficient and the Cronbach’s alpha coefficient for the scale in this study were both 0.88.

As shown in Table 3, six items with a factor loading above 0.6 were retained. The CR value was 0.88 and the AVE was 0.54. A good model fit was found for DDI in the CFA: *χ*^2^/DF = 4.102, *p* = 0.000, GFI = 0.983, AGFI = 0.959, CFI = 0.986, TLI = 0.977, IFI = 0.986, RMSEA = 0.065 [0.044, 0.088], and SRMR = 0.025.

- Altruistic tendency in everyday moral decision-making. The 24-item scenarios of everyday moral decision-making (see Appendix A Table A1 and Table A2), developed by Study 1, was used to dissociate individuals’ altruistic tendencies in everyday moral decision-making. The material consists of six scene frameworks, and each frame contains four scenarios representing the four dimensions of everyday moral decision-making dilemmas (egoistic and non-altruistic, egoistic and altruistic, non-egoistic and altruistic, and non-egoistic and non-altruistic). Four probability data exist for each dimension separately: p1 (egoistic and non-altruistic), p2 (egoistic and altruistic), p3 (non-egoistic and altruistic), and p4 (non-egoistic and non-altruistic), representing the average degree of decision makers’ approval of the proposed behavior. After obtaining the probability data, the CAN algorithm [7] was applied to calculate the three parameters representing individuals’ everyday moral decision-making tendencies: ET, AT, and OP. The AT was then used as the dependent variable in this study. The validity of this material was tested in Study 1.

#### 3.1.3. Procedure

The electronic questionnaire, titled State of Life and Scenario Decision-making Survey, was divided into four parts (all participants filled out an informed consent form on the first page of the questionnaire). The first part was a scenario decision-making task in which participants were told to imagine themselves as the main character in the scenarios and make a choice based on their first instinct after reading each scenario. The participants were asked to make a dichotomous choice for each scenario they read. An example is:

- You are eating at a restaurant and you see a person smoking at the table. If you walk over to him/her and remind him/her not to smoke in public, he/she is offended by your stopping him/her and may get into a physical confrontation with you at any time, but other diners will get a fresh dining environment. You choose to (single-choice): A. stop the smoker B. do not stop the smoker).

As in Study 1, the order of presentation of the material was pre-randomly controlled to ensure that two adjacent segments were from different dilemmas. Question 19 was used to check instructional manipulation (i.e., In order to confirm that the respondent has read the material carefully, this question is an instructional manipulation check. Please select both A and B options for this question.). The second and third parts examined participants’ distress disclosure and social isolation, respectively. The fourth part contained basic demographic information.

#### 3.1.4. Analytical Strategy

After the questionnaire was collected, the participants who passed the instructional manipulation check were screened out. All valid data were imported into SPSS 25.0 for testing the common method bias, analyzing the bivariate correlation between variables and differences in demographic variables for the three parameters (i.e., ET, AT, and OP).

### 3.2. Results

#### 3.2.1. Common Method Bias Analysis

The issue of common method bias was controlled for and tested, since the data for this study were collected through self-reporting. For procedural control, one instructional manipulation check was inserted with reference to Oppenheimer and Davidenko [27]. For statistical control, the Harman one-way test was conducted [31,32]. The unrotated exploratory factor analysis extracted 15 factors with eigenvalues exceeding one. The first factor explained 15.44% of the total variance, which was much less than the critical value of 40% [31], indicating that there were no significant common method variances in this study.

#### 3.2.2. Correlational Analysis

Table 4 presents the means and standard deviations of the variables, as well as the Pearson’s correlation results among the main research variables. In addition to including three dissociated parameters, altruistic choice (AC) probability was also calculated (i.e., the altruistic and non-egoistic tendency of individuals, expressed as p3 in Table 1). In previous research, researchers usually considered AC as an indicator to demonstrate people’s altruistic inclinations at a cost to egoistic interests [33]. With our process dissociation model, we can measure the AT, ET, and OP underlying the AC.

The results showed that SI is not significantly correlated with AC (*r* = 0.05, *p* = 0.183). However, with deeper insight by the CAN algorithm, we found it positively correlated with AT, *r* = 0.27, *p* < 0.001) and negatively correlated with OP (*r* = −0.11, *p* < 0.001). In other words, individuals with a higher sense of social isolation are more likely to behave altruistically and are less inclined to accept the given act in a specific context.

Similarly, DD has no significant correlation with AC (*r* = −0.01, *p* = 0.868). However, with the help of the CAN algorithm, we found it positively correlated with both AT (*r* = 0.19, *p* < 0.001) and ET (*r* = 0.07, *p* = 0.045), and negatively correlated with OP (*r* = −0.14, *p* < 0.001). That is, people who disclose distress to others more frequently have stronger altruistic tendency and egoistic tendency simultaneously and are less likely to accept the given act in a specific context.

In addition, the data showed a slightly positive correlation between age and AC (*r* = 0.09, *p* = 0.018). Further dissociation of psychological processes demonstrated only a negative relationship between age and ET (*r* = −0.16, *p* < 0.001). The results mean that the older the participants, the less egoistic they are.

#### 3.2.3. Individual Differences: Comparing Measured Variables by the Gender and Intimate Relationship

To determine whether males and females differ on the variables we are interested in, independent *t*-tests were conducted for all variables. The results in Table 5 showed a marginally significant difference in AC (*t* = 1.88, *p* = 0.061) between males and females. Further dissociation of the psychological processes based on the CAN algorithm supported higher AT (*t* = −2.94, *p* = 0.003) in females and higher OP (*t* = 2.25, *p* = 0.025) in males.

We also conducted independent *t*-tests for differences in participants’ intimacy status. As shown in Table 6, no significant intimacy difference in AC (*t* = −1.65, *p* = 0.100) was found. Yet, the intimacy differences were reflected in the egoistic tendencies that we dissociate through the CAN algorithm. Specifically, AT (*t* = 2.30, *p* = 0.022) was significantly stronger for single individuals than for non-single individuals.

### 3.3. Discussion

Consistent with the hypotheses, findings in Study 2 demonstrated that people’s social isolation and distress disclosure are positively related to their altruistic tendencies. In other words, the correlation between social isolation, distress disclosure, and traditional altruistic tendencies can be further dissociated into the correlation between social isolation, distress disclosure and altruistic tendencies, egoistic tendencies, and overall action/inaction preferences. To some extent, this result supports our dissociation of the psychological process of an individual’s decision-making and is conducive to providing a further insight on the multiple psychological processes.

## 4. General Discussion

The present research developed 52 scenario materials. After screening statistically in Study 1, 24 scenarios with high reliability and validity were obtained to constitute the everyday moral decision-making material. In Study 2, we applied the CAN algorithm to dissociate people’s process of making moral decisions in everyday life. We found that social isolation and distress disclosure are positively linked to an individual’s altruistic tendency. The results provided insight into the related variables that influence decision-making in altruistic versus egoistic moral dilemmas.

### 4.1. The Application of CAN Algorithm

The everyday moral decision-making paradigm has been widely accepted and used in the field of moral decision-making. However, the measurement of decision-making tendencies in previous studies has been limited to two situations: non-egoistic and altruistic and egoistic and non-altruistic. Once people choose to act altruistically, they are considered to be non-egoistic, which is somewhat ambiguous in the interpretation of the results. In particular, the overall action/inaction preference is ignored in such classifications of situations. For example, in the case of necessities shortage, you choose to exchange the only meat you have left for a neighbor’s noodles to satisfy his/her need for meat. This does not necessarily mean that you are non-egoistic. There is other possibilities, for example, you generally tend to accept or reject the request of others, whether in a barter situation or any other specific situation.

To address this issue, we applied the CAN algorithm [7] to dissociate the psychological process in moral decision-making through a comprehensive examination of the two conflicting parameters of altruistic (altruistic: altruistic/non-altruistic) and egoistic (egoistic/non-egoistic). This provides a methodological basis for further testing of the related variables of altruistic tendencies. 

### 4.2. The Relationship between Social Isolation, Distress Diclosure, and Altruistic Tendency in Everyday Moral Decision-Making

Previous studies have found a positive correlation between altruism and social integration or social connection [16,17]. However, the present study supports that altruistic tendency in everyday moral decision-making can also be positively associated with social isolation. 

According to the moral credits model, there is a bank account in an individual’s cognition, where compliance with norms is equivalent to making deposits and violation of norms means making withdrawals [34]. By obeying group norms for a long time, individuals accumulate special credibility that can be used to buy the right to violate group norms [35]. For example, a member who adheres to group norms over time is likely to do something that violates norms without considering himself/herself bad. An increase in an individual’s sense of social isolation leads to a decrease in the deposit in his/her psychological account as high social isolation deviates from the norms perceived by society at large. This is especially true for people with a Chinese cultural background; that is, isolation from the group means a violation of social values to some extent [36,37,38]. As a result, the individual’s sense of deficit in the mental account increases. This, in turn, motivates individuals to engage in behaviors that enhance social connection in order to gain belongingness. In this case, people are more inclined to choose to help others in moral dilemmas. Moreover, as outlined by social exchange theory, altruistic behavior can induce a sense of satisfaction in the helper. This intrinsic reward mechanism increases the level of self-affirmation and contributes to a positive level of psychological well-being [39,40]. At this level, helping others may indeed be a preferred behavior when individuals with high social isolation are faced with a moral dilemma.

Social penetration theory suggests that when motivated by the pursuit of group identity and social connection, individuals may resort to a basic form of social exchange and self-disclosure, which is the voluntary and truthful presentation of feelings and thoughts to others [41]. In this way, the individual receives interpersonal support and is thus able to release stress and better adapt to social life. Furthermore, individuals can gain self-identity and group identity from the object of disclosure, which encourages them to extend the reciprocity effect to other groups. Therefore, when faced with moral dilemmas in everyday life, people with a higher level of distress disclosure tend to behave altruistically (rather than egoistically), compared with those who are less likely to express their distress to others.

## 5. Contributions and Limitations

The current research provides important contributions. First, we draw on the CAN algorithm to develop everyday moral decision-making scenarios that are appropriate to the Chinese cultural background and further extend the algorithm to the field of altruistic vs. egoistic social dilemma research. Such an application dissociates people’s tendencies in everyday moral decision-making, which achieves a relatively powerful explanation theoretically. Thus, this study provides materials and methodological reference for similar studies in the future. Second, through empirical examination, we confirm the positive relationship between social isolation, distress disclosure, and altruistic tendency in everyday moral decision-making. These findings could deepen our understanding of how social isolation is related to altruistic tendency.

Several limitations should also be acknowledged. First, the characters involved in the development of scene material encompassed those people that they might encounter in their everyday social interactions (including friends, family, colleagues, neighbors, strangers, and net users). However, the number of scenarios corresponding to different characters was not completely consistent, since the scene frameworks were screened and eliminated after the Study 1. In the future, consideration could be given to developing more scenarios for screening to improve the consistency of the number of items for each character, thus improving the applicability of the material in real life. Next, the high similarity among decision-making scenarios requires participants to consume many cognitive resources to fill out the questionnaire. The resulting fatigue effect may affect survey results and the efficiency of the questionnaire. Future research could streamline the length of scenarios and reduce the burden on participants to fill out the questionnaire. Finally, since the characteristics (altruism or egoism) of the behavior measured in this study is sensitive in everyday moral decision-making, respondents are susceptible to social desirability [42]. In particular, the social desirability may be greater when the participants are close to the features of the character (e.g., the participant himself/herself was once a blogger who made a mistake in a published post). Future studies may consider adding a social desirability scale to the survey to improve the validity of the data.

## 6. Conclusions

Based on the validated self-developed material and the CAN algorithm, this study examined the psychological processes and the related variables of decision-making in everyday moral dilemmas. Our work provided a methodological protocol for future research on everyday moral decision-making.

## Figures and Tables

**Table 1:** An example scene framework.

Egoistic		Non-Egoistic	
Non-Altruistic	Altruistic	Altruistic	Non-Altruistic
p1	p2	p3	p4
You are eating at a restaurant and you see a person smoking at the table. If you leave alone, you can have a fresh dining environment without any conflict with the smoker, but other diners will continue to inhale secondhand smoke.	You are eating at a restaurant and you see a person smoking at the table. Others in the restaurant did not try to stop him/her. If you stop the smoker, he/she will extinguish the cigarette as a sign of apology, and everyone will praise you for your behavior.	You are eating at a restaurant and you see a person smoking at the table. If you walk over to him/her and remind him/her not to smoke in public, he/she is offended by your stopping him/her and may get into a physical confrontation with you at any time, but other diners will get a fresh dining environment.	You are eating at a restaurant and you see a person smoking at the table. Others in the restaurant did not try to stop him/her. If you choose to remain silent, you and other diners will continue to inhale secondhand smoke and suffer health consequences.

Note. p1, p2, p3, and p4 mean the probability of participants agreeing the proposed behavior in respective types of scenarios.

**Table 2:** Results of chi-square test of participants’ judgments on 13 frameworks, including 52 Scenarios (*n* = 209).

Scene Frameworks ^a^	Egoistic				Non-Egoistic			
	Non-Altruistic		Altruistic		Altruistic		Non-Altruistic	
	p1		p2		p3		p4	
	*n* ^b^ (%)	χ^2^	*n* ^b^ (%)	χ^2^	*n* ^b^ (%)	χ^2^	*n* ^b^ (%)	χ^2^
**Friend 1**	178 (85)	103.39 ***	124 (59)	7.28 **	131 (63)	13.44 ***	117 (56)	2.99
Friend2	131 (63)	13.44 ***	181 (87)	112.01 ***	151 (72)	41.38 ***	111 (53)	0.81
Relative 1	102 (49)	0.12	168 (80)	77.17 ***	134 (64)	16.66 ***	145 (69)	31.39 ***
**Relative 2**	116 (56)	2.53	181 (87)	112.01 ***	158 (76)	54.78 ***	128 (61)	10.57 ***
Colleague 1	58 (28)	41.38 ***	172 (82)	87.20 ***	154 (74)	46.90 ***	120 (57)	4.60 *
**Colleague 2**	133 (64)	15.55 ***	160 (77)	58.95 ***	130 (62)	12.45 ***	124 (59)	7.28 *
Neighbor 1	119 (57)	4.02 *	184 (88)	120.96 ***	152 (73)	43.18 ***	64 (31)	31.39 ***
Neighbor 2	146 (70)	32.96 ***	162 (78)	63.28 ***	143 (68)	28.37 ***	104 (50)	0.01
**Stranger**	118 (56)	3.49 *	175 (84)	95.12 ***	139 (67)	22.78 ***	126 (60)	8.85 ***
Supermarket staff	104 (50)	0.01	182 (88)	117.00 ***	95 (45)	1.73	128 (61)	10.57 ***
**Salesperson**	145 (69)	58.95 ***	185 (89)	124.02 ***	153 (73)	45.02 ***	117 (56)	2.99
**Blogger**	125 (60)	8.04 ***	181 (87)	112.01 ***	145 (69)	31.39 ***	133 (64)	15.55 ***
Online purchase	94 (45)	2.11	166 (79)	72.39 ***	102 (49)	0.12	62 (30)	34.57 ***

Note. Friend 1 = a friend asks to board a pet at your home, Friend 2 = a sick friend needs your care, Relative 1 = a relative asks you to borrow money, Relative 2 = a relative asks you to be a tour guide for a trip, Colleague 1 = a colleague asks you to do works on his/her behalf, Colleague 2 = a colleague faces an examination, Neighbor 1 = a neighbor practices piano out loud, Neighbor 2 = a neighbor asks you to collect delivery for him/her, Stranger = a stranger is smoking in public, Supermarket staff = a supermarket staff neglects his/her duties, Salesperson = a salesperson asks you to sign up for a membership, Blogger = a blogger made a mistake in the post, Online purchase = an online purchase needs your evaluation. ^a^ The black bolded font in the scene frameworks is the final retained frames. In the Relative 2 frame, the rate of agreement between participants’ evaluations and the design was 56% in the egoistic and non-altruistic scenario, and the chi-square test was close to significant. This frame was retained to minimize response bias by keeping the number of decision responses of participants to at least 24, according to Gawronski et al. [4]. ^b^ *n* indicates the number of participants who judged the frame to be consistent with the intended structure. * *p* < 0.05, ** *p* < 0.01, *** *p* < 0.001 (two-tailed). The same hereinafter.

**Table 3:** Standardized item loadings, CR, AVE, McDonald’s ω, and Cronbach’s α of measurement model.

Factor	Item	Standardized Item Loading	CR	AVE	McDonald’s ω	Cronbach’s α
Friendship Scale	FS1	0.724	0.82	0.51	0.88	0.88
	FS2	0.517				
	FS3	0.538				
	FS4	0.802				
	FS5	0.828				
Distress Disclosure Index	DDI1	0.626	0.88	0.54	0.82	0.81
	DDI2	0.660				
	DDI3	0.695				
	DDI4	0.838				
	DDI5	0.817				
	DDI6	0.759				

Note. FS = Friendship Scale, DDI = Distress Disclosure Index.

**Table 4:** Mean, standard deviation, and correlation coefficient of measured variables (*n* = 734).

Variables	M ± SD	1	2	3	4	5	6
1. SI	18.74 ± 4.17	1.00					
2. DD	17.42 ± 5.20	0.48 ***	1.00				
3. AT	0.22 ± 0.25	0.27 ***	0.19 ***	1.00			
4. ET	0.31 ± 0.23	0.06	0.07 *	−0.12 **	1.00		
5. OP	0.57 ± 0.11	−0.11 **	−0.14 ***	−0.07	−0.27 ***	1.00	
6. AC	0.46 ± 0.18	0.05	−0.01	0.55 ***	−0.68 ***	0.61 **	1.00
7. Age	26.66 ± 6.53	−0.00	−0.01	−0.04	−0.16 ***	0.06	0.09 *

Note. SI = social isolation, DD = distress disclosure, AT = altruistic tendency, ET = egoistic tendency, OP = overall action/inaction preference. AC = altruistic choice probability in the non-egoistic and altruistic scenario, i.e., p3 in Table 1. * *p* < 0.05, ** *p* < 0.01, *** *p* < 0.001 (two-tailed). The same hereinafter.

**Table 5:** Gender differences in measured variables (*n* = 734: male = 309, female = 425).

Variables	Male (M ± SD)	Female (M ± SD)	*t*	Sig.	Cohen’s d
SI	18.84 ± 4.02	18.67 ± 4.27	0.56	0.578	0.042
DD	17.2 ± 5.22	17.57 ± 5.19	−0.95	0.342	−0.071
AT	0.21 ± 0.26	0.23 ± 0.25	−0.86	0.392	−0.064
ET	0.28 ± 0.23	0.33 ± 0.22	−2.94 **	0.003	−0.220
OP	0.58 ± 0.09	0.57±0.12	2.25 *	0.025	0.168
AC	0.53 ± 0.24	0.49 ± 0.25	1.88	0.061	0.140

Note. * *p* < 0.05, ** *p* < 0.01 (two-tailed).

**Table 6:** Differences of intimate relationship in measured variables (*n* = 734: single = 358, non-single = 376).

Variables	Single (M ± SD)	Non-Single (M ± SD)	*t*	Sig.	Cohen’s d
SI	18.51 ± 4.23	18.96 ± 4.10	−1.46	0.144	−0.108
DD	17.36 ± 5.09	17.47 ± 5.32	−0.28	0.780	−0.021
AT	0.22 ± 0.23	0.22 ± 0.27	−0.03	0.973	−0.002
ET	0.33 ± 0.23	0.29 ± 0.22	2.30 *	0.022	0.170
OP	0.57 ± 0.11	0.58 ± 0.11	−0.89	0.376	−0.065
AC	0.49 ± 0.24	0.52 ± 0.25	−1.65	0.100	−0.121

Note. * *p* < 0.05 (two-tailed).

## Data Availability

The data that support the findings of this study are available upon request from the corresponding author.

## Appendix A

**Table A1:** The Six Frameworks with a Total of 24 Scenarios Retained in Study 1 (in English).

Scene Framework	Egoistic		Non-Egoistic	
	Non-Altruistic	Altruistic	Altruistic	Non-Altruistic
	p1	p2	p3	p4
1. A friend asks to board a pet at your home	A friend is planning a business trip and would like you to help him/her take care of a pet. If you stick to your travel schedule this week, your friend’s pet cannot be properly taken care of, but you can enjoy your vacation. (You choose to: A. take care of the pet B. do not take care of the pet)	A friend is planning a business trip and wants you to take care of his/her pet. You happen to have the weekend off and like the pet. If you take care of the pet for him/her, the friend will be able to travel with peace of mind and bring you a gift when he/she returns home as a token of appreciation. (You choose to: A. take care of the pet B. do not take care of the pet)	A friend is planning a business trip and would like you to help him/her take care of a pet. If you agree to help, the friend can travel with peace of mind. However, this may take some of your time off, and the pet is a bit naughty and may cause damage to your furniture. (You choose to: A. take care of the pet B. do not take care of the pet)	A friend is planning a business trip and wants you to take care of his/her pet for a fee. If you stick to your weekend off schedule, the pet will not be taken care of, and you will lose this income that can solve your urgent needs. (You choose to: A. take care of the pet B. do not take care of the pet)
2. A relative asks you to be a tour guide for a trip	A relative is on a trip to your city. He/she asks you to act as a tour guide because he/she is not familiar with the place. If you stick to your schedule and do not take him/her on the tour, he/she will probably not have a good trip, but you will have more rest time. (You choose to: A. stick to your schedule and do not take him/her on the tour B. adjust your schedule and take him/her on the tour)	A relative is on a trip to your city. He/she asks you to act as a tour guide because he/she is not familiar with the place. If you take him/her on a tour, the relative will have a smooth journey, and you can also have a chance to take a break and relieve the strain of work. (You choose to: A. take him/her on a tour B. do not take him/her on a tour)	A relative is on a trip to your city. He/she asks you to act as a tour guide because he/she is not familiar with the place. If you take him/her on a tour, he/she will have a smooth journey, but it will disrupt your rest schedule. (You choose to: A. take him/her on a tour B. do not take him/her on a tour)	A relative is on a trip to your city. He/she asks you to act as a tour guide because he/she is not familiar with the place. If you stick to your schedule and do not take him/her on the tour, he/she will probably not have a good trip, and your indifferent treatment may be known by other relatives, causing you to lose their concern for you and your family. (You choose to: A. stick to your schedule and do not take him/her on the tour B. adjust your schedule and take him/her on the tour)
3. A colleague faces an examination	You are an employee of the human resources of a company. You receive a notice from your supervisor that a random inspection will be conducted tomorrow in department A to punish employees who fail the inspection. You are asked to keep this inspection to yourself. A colleague with whom you have a good relationship happens to work in department A. If you keep the news, you will be rewarded by the supervisor for performing your duties properly. However, your colleagues may be punished for failing the inspection. (You choose to: A. keep the news B. break the news to him/her)	You are an employee of the human resources of a company. You receive a notice from your supervisor that a random inspection will be conducted tomorrow in department A to punish employees who fail the inspection. A colleague with whom you have a good relationship happens to work in department A. If you break the news to him/her, he/she will be ready to pass the spot check perfectly. Also, you will get stronger support from him/her in your future work. (You choose to: A. break the news to him/her B. do not break the news to him/her)	You are an employee of the human resources of a company. You receive a notice from your supervisor that a random inspection will be conducted tomorrow in department A to punish employees who fail the inspection. You are asked to keep this inspection. A colleague with whom you have a good relationship happens to work in department A. If you break the news to him/her, he/she will be ready to pass the spot check perfectly. However, you will be punished for violating your duties once your supervisor learns that you leaked the information. (You choose to: A. break the news to him/her B. do not break the news to him/her)	You are an employee of the human resources of a company. You receive a notice from your supervisor that a random inspection will be conducted tomorrow in department A to reward employees with excellent appraisals. A colleague with whom you have a good relationship happens to work in department A. If you keep the news to yourself, he/she will miss this opportunity for promotion, and you will be complained about by him/her and lose his/her support in the future. (You choose to: A. keep the news B. break the news to him/her)
4. A stranger is smoking in public	You are eating at a restaurant and you see a person smoking at the table. If you leave alone, you can have a fresh dining environment without any conflict with the smoker, but other diners will continue to inhale second-hand smoke. (You choose to: A. leave alone B. do not leave alone)	You are eating at a restaurant and you see a person smoking at the table. Others in the restaurant did not try to stop him/her. If you stop the smoker, he/she will extinguish the cigarette as a sign of apology, and everyone will praise you for your behavior. (You choose to: A. stop the smoker B. do not stop the smoker)	You are eating at a restaurant and you see a person smoking at the table. If you walk over to him/her and remind him/her not to smoke in public, he/she is offended by your stopping him/her and may get into a physical confrontation with you at any time, but other diners will get a fresh dining environment. (You choose to: A. stop the smoker B. do not stop the smoker)	You are eating at a restaurant and you see a person smoking at the table. Others in the restaurant did not try to stop him/her. If you choose to remain silent, you and other diners will continue to inhale second-hand smoke and suffer health consequences. (You choose to: A. leave alone B. do not leave alone)
5. A salesperson asks you to sign up for a membership	You are shopping when a salesperson stops you and asks you to sign up for a free membership to a restaurant. This requires you to fill in personal information (e.g., name and phone number). If you do not sign up for the membership, this salesperson will not complete the task on time, but you will avoid the risk of personal information being leaked. (You choose to: A. not sign up for the membership B. sign up for the membership)	You are shopping when a salesperson stops you and asks you to sign up for a free membership to a restaurant. You happen to be interested in this restaurant. If you sign up for membership, you will get a coupon for the restaurant and help the salesperson to get the job done. (You choose to: A. sign up for the membership B. not sign up for the membership)	You are shopping when a salesperson stops you and asks you to sign up for a free membership to a restaurant. This requires you to fill in personal information (e.g., name and phone number). If you sign up for a membership, you can help this salesperson get the job done as quickly as possible, but your personal information may be leaked. (You choose to: A. sign up for the membership B. not sign up for the membership)	You are shopping when a salesperson stops you and asks you to sign up for a free membership to a restaurant you are interested in. If you do not sign up for the membership, you will not be able to enjoy the discounts of that restaurant and this salesperson will not be able to complete the task on time. (You choose to: A. not sign up for the membership B. sign up for the membership)
6. A blogger made a mistake in the post	A blogger you followed published an article, and you find that some of its details violate the rules of the social platform. If you report to the platform about this blogger, he/she cannot earn income from posting over a period, but you will get the rewards from the platform. (You choose to: A. report to the platform B. do not report to the platform)	A blogger you followed published an article and you find some mistakes in the details. If you contact him/her privately to point out these mistakes, he/she will fix the details and avoid making a big mistake, and you will get a gift of gratitude from him/her. (You choose to: A. point out the mistakes B. do not point out the mistakes)	A blogger you followed published an article and you find some mistakes in the details. If you leave a comment pointing out these mistakes, he/she will fix the details and avoid making a big mistake, but you will be abused by his/her fans (for damaging the blogger’s reputation). (You choose to: A. point out the mistakes B. do not point out the mistakes)	A blogger you followed published an article and you find some mistakes in the details. If you leave a comment pointing out these mistakes, he/she will be accused of ignorance, and you will be abused by his/her fans (for damaging the blogger’s reputation). (You choose to: A. point out the mistakes B. do not point out the mistakes)

**Table A2:** The Six Frameworks with a Total of 24 Scenarios Retained in Study 1 (in Chinese).

情境框架	利己		不利己	
	不利他	利他	利他	不利他
	p1	p2	p3	p4
**1. 朋友的宠物需要寄养**	你的好朋友周末要出差，请你照顾ta养的一只宠物。如果你坚持这周的出游安排，朋友的宠物无法得到妥善照顾，但你可以享受自己的假期。 （你选择：A. 坚持出游安排, 不替ta照顾宠物 B.放弃出游安排，替ta照顾宠物）	你的好朋友周末要出差，请你照顾ta养的一只宠物。刚好你周末休息，同时也很喜欢这只宠物，如果替ta照顾，那么朋友能安心出差，回家后也会给你带一份伴手礼以示感激。 （你选择：A.替ta照顾宠物 B.不替ta照顾宠物）	你的好朋友周末要出差，请你照顾ta养的一只宠物。如果选择替ta照顾宠物，朋友能安心出差，但会占用你休息的时间，并可能给你的家具造成一些破坏。 （你选择：A.替ta照顾宠物 B.不替ta照顾宠物）	你的好朋友周末要出差，因此请你照顾ta养的一只宠物，并打算付给你一定酬劳。如果你坚持周末休息，不替朋友照顾宠物，那么朋友不能安心出差，你也会失去这笔能解决燃眉之急的收入。 （你选择：A.替ta照顾宠物 B.不替ta照顾宠物）
**2.亲戚需要导游**	家一位亲戚来到你所在的城市旅游，ta因为人生地不熟，所以请你充当ta的导游。如果你坚持自己的生活安排，不带ta游玩，ta这趟旅游必然会不那么顺利，并且会遭遇骗子，但你将拥有更多休息时间。 （你选择：A.坚持自己的安排，不带ta游玩 B.调整自己的安排，带ta游玩）	老家一位亲戚来到你所在的城市旅游，ta因为人生地不熟，所以请你充当ta的导游。如果带ta游玩，亲戚能玩得更顺利，以后更加关照你，你也可以借此机会散散心，缓解工作的劳累。 （你选择：A.带ta游玩 B.不带ta游玩）	老家一位亲戚来到你所在的城市旅游，ta因为人生地不熟，所以请你充当导游带ta游玩几天，这样ta能玩得更顺利，但会打乱你下班后的休息安排。 （你选择：A.带ta游玩 B.不带ta游玩）	老家一位亲戚来到你所在的城市旅游，ta因为人生地不熟，所以请你充当ta的导游。如果你坚持生活安排，不带ta游玩，亲戚这趟旅游必然不会那么顺利，并且可能遭遇骗子，而你的“冷漠对待”可能传到其他亲戚耳中，从而使你失去他们的关照。 （你选择：A.坚持自己的安排，不带ta游玩 B.调整自己的安排，带ta游玩）
**3. 同事面临考核**	你是某公司的HR，今天你收到主管通知，明天将对A部门进行突击抽查考核，惩罚考察不合格的员工，并且要求你对这次考核保密。与你关系很好的一位同事刚好在A部门工作。如果你保守考核秘密，那么你会因正常履行了自己的工作职责而受到领导嘉奖，但同事可能因为考察不合格而受到惩罚。 （你选择：A.保守秘密，不告诉ta考核的消息 B.告诉ta考核的消息）	你是某公司的HR，今天你收到主管通知，明天将对A部门进行突击抽查考核，惩罚考察不合格的员工。与你关系很好的一位同事刚好在A部门工作，如果你将考核的消息告诉ta，那么ta将做好准备，完美通过突击考察，并且你们的关系将更加亲密，你在以后的工作中也能获得更强有力的支持。 （你选择：A.告诉ta考核的消息 B.不告诉ta考核的消息）	你是某公司的HR，今天你收到主管通知，明天将对A部门进行突击抽查考核，惩罚考察不合格的员工，并且要求你对这次考核保密。与你关系很好的一位同事刚好在A部门工作，如果你将考核的事情告诉ta，那么ta将做好准备，完美通过突击考察，但你违反了自己的工作职责，并且一旦主管得知你泄露了消息，将因此处罚你。 （你选择：A.告诉ta考核的消息 B. 不告诉ta考核的消息）	你是某公司的HR，今天你收到主管通知，明天将对A部门进行突击抽查考核，对考核优秀的员工予以奖励。与你关系很好的一位同事刚好在A部门工作。如果你保守工考核秘密，不告诉ta考核的事情，那么ta将错过这个晋升的机会，你也会被ta埋怨，并在以后的工作中被ta穿小鞋。 （你选择：A.保守秘密，不告诉ta考核的消息 B.告诉ta考核的消息）
**4.陌生人公共场合吸烟**	你在餐厅吃饭时，看到一个人在餐桌前抽烟。如果你独自离开，可以获得一个清新的就餐环境，且不会和吸烟者发生任何冲突，但其他就餐者将继续吸入二手烟。 （你选择：A.独自离开 B.不独自离开）	你在餐厅吃饭时，看到一个人在餐桌前抽烟，餐厅里没有人去制止吸烟者。如果你去制止吸烟者，ta会掐灭烟卷以示歉意，大家都将拥有一个清新的就餐环境，并赞扬你的高素质行为。 （你选择：A.制止吸烟者 B.不制止吸烟者）	你在餐厅吃饭时，看到一个人在餐桌前抽烟。此时你走过去，提醒ta不要在公共场合吸烟，而ta对你的制止感到不快，随时会和你产生肢体冲突，但其他就餐者都会获得一个清新的就餐环境。 （你选择：A.制止吸烟者 B.不制止吸烟者）	你在餐厅吃饭时，看到一个人在餐桌前抽烟，餐厅里没有人去制止吸烟者。如果你选择沉默，那么你和周围人都将继续吸入二手烟，健康受到影响。 （你选择：A.制止吸烟者 B.不制止吸烟者）
**5. 推销人员请求注册会员**	逛街时，一名推销人员拦住你，请你免费注册某火锅店会员，并需要你填写姓名、电话号码等信息。如果你不注册会员，这名推销人员将需要更多时间来完成任务，但你不用花费时间填写信息，同时避免了隐私泄露的可能。 （你选择：A. 放弃优惠，不注册会员 B.注册会员）	逛街时，一名推销人员拦住你，请你免费注册某火锅店会员，刚好你对这家火锅店很感兴趣，注册会员后将获得该火锅店的优惠券，同时帮助这名推销人员完成工作任务。 （你选择：A.注册会员 B.不注册会员）	逛街时，一名推销人员拦住你，请你免费注册某火锅店会员，并需要你填写姓名、电话号码等信息。如果你注册会员，可以帮这名推销人员尽快完成任务，但你的个人信息可能会被泄露。 （你选择：A.注册会员 B.不注册会员）	逛街时，一名推销人员拦住你，请你免费注册某家你感兴趣的火锅店会员。如果你不注册会员，将无法享受该火锅店的折扣优惠，这名推销人员也不能尽早完成任务。 （你选择：A.放弃优惠，不注册会员 B.注册会员）
**6.举报某博主的文章错误**	你关注的一位博主发表了一篇文章，你发现其中有些细节错误违反了该社交平台的规定。如果你向平台投诉这位博主，那么ta在一段时间内不能发表文章，也无法取得相关收益，但你将获得平台的奖励。 （你选择：A.向平台投诉B. 不向平台投诉）	你关注的一位博主发表了一篇文章，你发现其中有些细节错误，如果你私聊ta指出错误之处，ta会与你讨论并修正文章，从而避免因小失大，同时给你寄送礼物以表感激。 （你选择：A.指出细节错误 B.不指出细节错误）	你关注的一位博主发表了一篇文章，你发现其中有些细节错误。如果你留言指出这些错误，ta将避免因小失大，但你会被ta的粉丝骂成杠精。 （你选择：A.指出细节错误 B.不指出细节错误）	你关注的一位博主发表了一篇文章，你发现其中有些细节错误。如果你直接在评论区留言指出这些错误，ta将因此被骂学识浅薄，而你也会被ta的粉丝骂成杠精。 （你选择：A.指出细节错误 B.不指出细节错误）
